# Dietary supplementation of micro-encapsulated sodium butyrate in healthy horses: effect on gut histology and immunohistochemistry parameters

**DOI:** 10.1186/s12917-020-02332-4

**Published:** 2020-04-28

**Authors:** W. A. Wambacq, D. A. van Doorn, P. M. Rovers-Paap, R. Ducatelle, L. Vlaminck, M. Lourenço, M. Hesta

**Affiliations:** 1 Laboratory of Animal Nutrition, Department of Nutrition, Genetics and Ethology, Faculty of Veterinary Medicine, Ghent University, Heidestraat 19, 9820 Merelbeke, Belgium; 2 Department of Equine Sciences and Department of Farm Animal Health, Faculty of Veterinary Medicine, Utrecht University, P.O. Box 80.151, 3508 TD Utrecht, the Netherlands; 3 Equivado, Equine Nutrition Consultancy, Marnixlaan 80, 3552 HG Utrecht, the Netherlands; 4 Orffa, Vierlinghstraat 51, 4251LC Werkendam, the Netherlands; 5 Department of Pathology, Bacteriology and Avian Diseases, Faculty of Veterinary Medicine, Ghent University, Salisburylaan 133, 9820 Merelbeke, Belgium; 6 Department of Surgery and Anesthesiology of Domestic Animals, Faculty of Veterinary Medicine, Ghent University, Salisburylaan 133, 9820 Merelbeke, Belgium; 7 Present address: Flanders Research Institute for Agriculture, Fisheries and Food, Scheldeweg 68, 9090 Melle, Belgium

**Keywords:** Butyrate, Diet, Equine, Histology, Immunohistochemistry

## Abstract

**Background:**

As colic and intestinal disorders are a major concern in horses, the aim of the present study was to investigate the effect of dietary supplementation of butyrate, known to have a diverse array of beneficial effects on intestinal health. The effect of micro-encapsulated sodium butyrate supplementation on gut histology and immunohistochemistry parameters was studied in 14 healthy warmblood horses destined for slaughter in two separate periods. Horses were fed a low fiber - high starch diet, designed to induce subsequent starch overflow in the large intestine, aiming to create a mild challenge for large intestinal health. Treatment included supplementation with either micro-encapsulated sodium butyrate (Excential Butycoat®, Orffa, Werkendam, the Netherlands) or placebo (containing only coating material). The horses were fed for 20 consecutive days at a dosage of 0.4 g/kg BW (body weight). At day 21, the horses were slaughtered and intestinal samples were collected for determination of gut pH, villus length, crypt depth and area % of CD3+ and CD20+ cells.

**Results:**

Horses on the butyrate supplemented diet had significantly reduced crypt depths in the right dorsal colon compared to placebo-fed horses (*P* < 0.001). However, a treatment x period interaction (*P* = 0.002) was discovered regarding this parameter, which could not be explained by the authors. Further investigation into the number of KI67+ cells in the RDC crypts did not reveal any significant differences between treatments (*P* = 0.650), indicating that the reduction in crypt depth in butyrate-fed horses could not be explained by a significant difference in cellular proliferation. Intestinal pH, villus length and expression of intestinal CD3+ and CD20+ cells were not significantly affected by treatment at any intestinal level.

**Conclusions:**

Our data indicate that supplementation of micro-encapsulated sodium butyrate to the equine diet did not influence gut histology (with the exception of a decrease found in the crypts of the RDC) or immunohistochemistry parameters in healthy horses. Further research is warranted to investigate the impact of butyrate supplementation in horses with intestinal disease.

## Background

Gastrointestinal health is an important topic in equine practice, as colic remains to this day one of the major causes of death in horses [1]. Feeding high-starch diets to horses is considered in conflict with the natural feed intake behavior but still common in practice [2]. Including high amounts of cereals or concentrate inclusion may result in undigested starch reaching the hindgut, increasing the risk for microbiome dysbiosis and subsequently colic [3]. Colic and intestinal disorders are also known to influence gut wall integrity [4], and can cause alterations such as mucosal degeneration in case of intestinal strangulation [5]. The objective of the current study was to investigate whether a dietary supplement could counteract the potential negative health effects on gut wall dynamics that have been associated with the feeding of high-starch diets. Research in other animal species has shown that increasing the concentration of the short chain fatty acid butyric acid in the intestinal lumen results in a diverse array of beneficial effects on intestinal health [6]. This end-product of microbial fermentation of fiber was previously described as the metabolic fuel of choice for colonocytes, resulting in increased colonocyte differentiation in both in vitro and in vivo studies in rats [7, 8]. In addition, there is evidence that butyric acid has an anti-inflammatory effect [9, 10], can modulate oxidative stress [11, 12], increase intestinal blood flow [13], and decrease the proliferation and differentiation of tumor cell lines [8]. Therefore, the aim of the present study was to investigate the effects of dietary supplementation of micro-encapsulated sodium butyrate on gut pH, villus length, crypt depth and CD3 and CD20 expression in healthy adult horses subjected to a high starch diet. This is the first study investigating the effect of sodium butyrate supplementation in the equine species.

## Results

### Feed intake, body weight, fecal pH and fecal consistency during supplementation period

Diets were well tolerated and none of the horses showed signs of illness, except for two horses that experienced hyporexia (for one and 3 days, respectively). Feed intake, body weight and fecal parameters are shown in Tables 1 and 2. There was no significant effect of time nor treatment on feed intake in all horses. A significant (*P* < 0.001) increase in body weight was observed during the course of the study for both groups, without statistical differences between the two dietary groups (*P* = 0.164). An average daily decrease of 0.013 pH units was observed in fecal pH from placebo-fed horses throughout the study (*P* = 0.05). There was no difference between dietary treatments in regard to fecal pH (*P* = 0.423). There was no significant effect of time nor treatment on fecal consistency throughout the study (*P* = 0.261 and *P* = 0.312, respectively).

**Table 1:** Feed composition and mean feed intake, bwt, fecal pH and consistency at day 1 and 20

Component	Haylage^a^		Concentrate^a,b^	
DM, g/kg product	861.6		895.4	
Crude ash, g/kg DM	59.3		63.0	
Crude fiber, g/kg DM	324.1		50.9	
Crude fat, g/kg DM	11.1		81.3	
Crude protein, g/kg DM	80.3		134.1	
Sugar, g/kg DM	137.1		60.5	
Starch, g/kg DM	0.0		351.2	
Component	Butyrate supplement		Placebo supplement	
Sodium, g/kg supplement	63.9		0.86	
Butyric acid, g/kg supplement	238		< 0.1	
Mean	Butyrate		Placebo	
	d1	d20	d1	d20
Roughage intake (kg)	6.9	6.9	6.9	6.9
Concentrate + supplement intake (kg)^c^	5.8	5.8	5.9	5.9
BW (kg)	559	579	571	586
Fecal pH	6.97	6.67	6.88	6.62
Fecal consistency score^d^	3	3	3	3

^a^ Analyzed by wet chemistry

^b^ Ingredient composition of the concentrate (Cavalor Mash & Mix^c^): flaked barley, wheat bran, linseed, oats, expanded barley, expanded corn, cane molasses, toasted soybeans, horse bean flakes, carrot pieces, calcium carbonate, leek, sodium chloride, soybean meal, fructo oligosaccharides, sunflower meal, palm kernel expeller

^c^ Concentrate and supplement provided together (mean values 5.6 kg concentrate + 229 g supplement in butyrate-fed group and 5.7 kg concentrate + 230 g supplement in placebo-fed group)

^d^ Fecal consistency score (1, watery feces, 2, decreased consistency, 3, ideal, 4: hard, 5: constipation)

**Table 2:** Longitudinal analysis of feed intake, bwt, fecal pH and fecal consistency

Coëfficient	value	SD	*P*-value
Concentrate + supplement intake (kg)^a^			
Day	0.000	0.040	0.991
Treatment	−0.171	0.855	0.841
Day x treatment	0.001	0.063	0.990
Roughage intake (kg)			
Day	0.000	0.015	0.991
Treatment	−0.003	0.320	0.992
Day x treatment	0.000	0.024	0.990
Total feed intake (kg roughage + concentrate + supplement)			
Day	−0.001	0.054	0.990
Treatment	−0.175	1.157	0.880
Day x treatment	0.001	0.085	0.990
Bodyweight (kg)			
Day	0.773	0.170	< 0.001
Treatment	−17.680	28.895	0.542
Day x treatment	0.331	0.236	0.164
Fecal pH			
Day	−0.013	0.005	0.005
Treatment	0.007	0.185	0.970
Day x treatment	−0.005	0.006	0.423
Fecal consistency (1–5 scale)			
Day	−0.007	0.006	0.261
Treatment	0.113	0.192	0.560
Day x treatment	−0.008	0.008	0.312

^a^ Interpretation of longitudinal analysis: Horses in the placebo group ate on average 5.9 kg concentrate mixed with placebo supplement a day over the course of the study. Horses fed the butyrate supplement ate 171 g less on average, but this was not significantly different (*P* = 0.841)

### Intestinal pH at slaughter

A period effect was found for CAE pH (*P* = 0.047) and LVC pH (*P* = 0.001). There was no effect of treatment for intestinal pH at all locations sampled (Table 3).

**Table 3:** pH, gut histology and immunohistochemistry parameters at different intestinal levels

	Butyrate		Placebo		*P* - value				
	Mean	SD	Mean	SD	Treatment	Period	Age	Sex	Treatment x Period
pH									
D	5.96	0.90	6.39	0.28	0.119	0.308	0.36	0.149	0.053
J	7.20	0.52	7.46	0.21	0.166	0.442	0.385	0.803	0.225
I	7.58	0.22	7.49	0.25	0.110	0.143	0.66	0.418	0.061
CAE	6.74	0.15	6.67	0.14	0.820	0.047	0.746	0.961	0.768
LVC	6.61	0.20	6.64	0.24	0.697	0.001	0.174	0.966	0.467
RDC	6.56	0.24	6.55	0.10	0.375	0.165	0.142	0.852	0.583
Crypt depth (μm)									
CAE	262	33	262	34	0.143	0.195	0.822	0.916	0.118
LVC	318	42	320	41	0.262	0.662	0.731	0.488	0.251
RDC	399	94	518	84	< 0.001	0.314	0.798	< 0.001	0.002
Area % of CD3 positive cells									
D	10.096	5.131	6.193	4.535	0.745	0.010	0.802	0.127	0.192
J	8.306	6.108	12.367	7.903	0.241	0.051	0.253	0.503	0.282
I	9.964	6.782	14.451	10.616	0.258	0.036	0.313	0.733	0.283
CAE	16.562	10.081	16.945	9.767	0.482	0.151	0.924	0.682	0.433
LVC	11.585	8.596	12.441	4.212	0.301	0.407	0.293	0.598	0.407
RDC	11.126	7.560	14.240	6.977	0.207	0.173	0.398	0.769	0.254
Area % of CD20 positive cells									
D	6.571	7.349	5.519	4.850	0.232	0.004	0.235	0.076	0.085
J	3.798	4.169	2.279	1.585	0.241	0.051	0.253	0.503	0.282
I	9.428	11.087	3.143	2.409	0.714	0.529	0.761	0.021	0.840
CAE	2.427	2.981	2.720	2.110	0.759	0.159	0.505	0.079	0.704
LVC	3.186	3.127	2.452	2.854	0.153	0.068	0.008	0.032	0.034
RDC	2.950	3.022	3.240	2.448	0.940	0.324	0.510	0.785	0.805
Number of KI67 positive cells per crypt									
RDC	25	10	32	12	0.650	0.984	0.687	0.632	0.972

*D* duodenum, *J* jejunum, *I* ileum, *CAE* cecum, *LVC* left ventral colon, *RDC* right dorsal colon

### Gut histology - intestinal villus length, crypt depth and villus length/crypt depth ratio

There was no effect of treatment regarding villus length nor villus length/ crypt depth ratio in all segments of the small intestine (data not shown). Horses on the butyrate supplemented diet had significantly lower crypt depths in the RDC compared to placebo-fed horses (*P* < 0.001) (Table 3). For this parameter, a sex- (*P* < 0.001) and a treatment x period effect was found (*P* = 0.002). A closer look at the data revealed a significant treatment effect in the first period (Mean diet B = 360 μm ± 24, Mean diet *P* = 559 μm ±52, *P* < 0.001) but not in the second (Mean diet B = 439 μm ± 121, Mean diet *P* = 463 μm ± 90, *P* = 0.821).

### Gut immunohistochemistry – area % of CD3 and CD20+ cells and KI67+ cell count

A significant period effect was found for the expression of CD3 positive cells in the duodenum and ileum (*P* = 0.010 and *P* = 0.036, respectively), whereas for the expression of CD20 positive cells, the period effect was only significant in the duodenum samples (*P* = 0.004). No treatment effect was found for intestinal expression of CD3 or CD20 positive cells in any segment of the gastro-intestinal tract. Expression of KI67 positive cells was not significantly different between treatments in the right dorsal colon crypts (Table 3).

## Discussion

A significant rise in body weight over the course of the study was detected in all horses as they were fed above maintenance energy requirements due to the formulated low fiber - high starch diet. The decrease in fecal pH in both butyrate and placebo supplemented groups throughout the supplementation period is likely due to the fact that the diets were rich in readily-fermentable starch. When this rapidly fermentable carbohydrate arrives in the hindgut, it favours the growth of bacteria that generate lactate, thereby leading to acidification of gut content and hence of feces [2].

The treatment x period interaction (*P* = 0.002) observed for the crypt depth in the RDC is difficult to explain. A decrease in the nutritive value of the forage due to storage [14] (the same forage batch was offered in the two experimental periods) could be a basis for this interaction. The reduction in RDC crypt depth in horses on the butyrate supplemented diet (*P* < 0.001) contradicts previous findings in other species, as butyrate supplementation is generally associated with an increase in crypt depth [6]. In fact, short-chain fatty acids are known to stimulate epithelial cell proliferation and differentiation, thereby improving healthy tissue turnover [15]. This indicates that findings in poultry, calves and piglets may not be translated directly to the equine species, as this specific hindgut fermenter’s gastrointestinal tract does not resemble that of the other species. Further investigation into the number of KI67+ cells in the crypts of the right dorsal colon segment in the current study did not reveal any significant differences between placebo and butyrate supplemented horses (*P* = 0.650), indicating the reduction in crypt depths from butyrate-fed horses could not be explained by a significant difference in cellular proliferation. However, increased crypt cell proliferation rate is not the only cause of an increase in crypt depth. Deeper crypts can be associated with decreased crypt cell apoptosis rate, cell hypertrophy, or a combination of these factors as well [16]. A similar reduction in crypt depth (although only a numerical difference) was reported at the level of the caecum, when supplementing 0.5 g sodium butyrate/kg diet to growing rabbits (also hindgut fermenters) [17]. However, crypt depths in the previous study increased numerically (but remained similar to when a control diet was fed) when the dosage of butyrate was increased to 1.0 and 2.0 g/kg diet compared to supplementation with 0.5 g sodium butyrate/kg diet. The amount of butyrate supplemented in the current study (0.4 g/kg BW or about 18 g/kg diet) was based on the study of Glinksy et al. [18], and provided a supplemental 20% of the estimated normal cecal butyric acid production rate. Dosage of butyrate supplementation likely plays an important role, and could explain the reduction in RDC crypt depth in our study.

With regard to the anti-inflammatory features of butyrate [9, 10], significant differences for the expression of CD3 and CD20 cells between the butyrate supplemented and the control horses could be expected. However, the expression of intestinal CD3 cells was not significantly affected by treatment in any of the segments of the gut studied, nor was this the case for the CD20 positive cell population.

A detailed investigation regarding the sodium butyrate release from the micro-capsules would have been valuable. Ideally, the micro-encapsulated sodium butyrate supplement would be released gradually in the large intestine. Indeed, coating particles were found upon visual inspection throughout all digestive tract segments of the horses. However, this does not necessarily indicate that the release of butyric acid happened gradually, since the degradation of coating could take longer than the absorption of butyrate. Considering the (mean) retention time of a typical horse diet [19], in combination with an in-house performed in-vitro release test of the supplement (unpublished observations) and the reported availability in other animal species [20], it seems likely that the butyric acid coated supplement would in fact be released gradually in the large intestine. However, it cannot be excluded that the supplement was not, or only partly, released in the large intestine.

The diet used in the current study was formulated to induce a certain amount of starch overflow into the large intestine by providing 2.5 g sugar and starch/kg BW/meal (1.6 g starch/kg BW/meal). It has been demonstrated that starch fed in excess of 2 g/kg BW/meal may result in potentially harmful quantities of starch reaching the hindgut in horses [3]. In order to prevent carbohydrate-overload induced hindgut disturbances (acidosis, diarrhea, colic) and associated problems (weight loss, laminitis), the authors chose not to formulate a diet containing a sugar and starch content that was extremely high. The purpose of the diet formulated in the present study was to induce subclinical disturbances, but to avoid clinical presentation of hindgut acidosis in the horses. It is however possible that our model did not induce enough acidification and inflammation in the hindgut of the horses, failing to demonstrate the beneficial effects of butyrate supplementation in subclinical gut inflammatory conditions. Furthermore, as this study was performed in slaughter horses, a cross-over design was not possible and could be identified as a weakness in the current study. Further studies should focus on horses suffering from large intestinal disease (e.g. right dorsal colitis, carbohydrate overload-induced diarrhea) in order to determine whether supplementing butyric acid can reverse these conditions.

## Conclusion

In conclusion, the present study indicates that supplementation of micro-encapsulated sodium butyrate at 0.4 g/kg BW to healthy adult horses on a low fiber – high starch diet did not significantly influence feed intake, faecal consistency, faecal or intestinal pH, villus length, crypt depth (with the exception of a decrease found in the crypts of the RDC) or intestinal expression of CD3, CD20 and KI67 (RDC) positive cells.

## Methods

### Animals and diet

Fourteen adult warmblood horses (Keros nv, Zonnebeke, Belgium), destined for slaughter, were included in the study and were allocated to one of two groups ensuring uniform distribution between groups of age, sex, body weight (BW) and body condition score (BCS) [21]. Each group contained six intact females and one gelding with a mean age of 10 ± 5.2 and 13 ± 3.1 years, a mean body weight of 571 ± 49.1 and 559 ± 74.5 kg, and a mean BCS of 4/9 ± 1.1 and 4/9 ± 1.0, for the placebo and butyrate supplemented group, respectively. There was no statistical power analysis performed to demonstrate that the quantity of animals used in the study would correspond with the quantity that is statistically required to obtain scientifically relevant results. As this is the first study examining the effect of butyrate supplementation in horses, ​​the standard deviation for the parameters to be examined in horses and the magnitude of the expected effect was unknown at the time. A thorough clinical examination was performed in all horses prior to the study demonstrating all animals were healthy. This examination included a dental check-up, blood- and fecal analysis. None of the horses had a history of gastro-intestinal disorders. Prior to the study, all horses were dewormed using an oral gel preparation containing 200 μg ivermectine and 1.5 mg praziquantel/kg BW (Equest pramox, Fort Dodge Animal Health Benelux B.V., Weesp, the Netherlands). The horses were individually housed in stables (3x5m) with wood shavings as bedding. They were allowed access to a sand paddock for a few hours three times a week. All horses were fed a low fiber – high starch diet designed to induce subsequent starch overflow to the large intestine, thereby aiming to create a mild challenge for large intestinal health. This diet consisted of haylage (given at 1% of ideal BW on DM basis) and a mash concentrate (Cavalor Mash and Mix, Cavalor, Belgium), and provided 2.5 g sugar and starch/kg BW per meal (Table 1). Horses were consequently fed well above NRC daily maintenance energy requirements [22]. The diet met NRC [22] vitamin and mineral requirements and was divided into two equal meals given at 0800 and 1700 h. Water remained available at all times.

### Experimental design

The experimental protocol was approved by the Ethical Committee of the Faculty of Veterinary Medicine, Ghent University, Belgium (EC 2014/103) and was in accordance with national and institutional guidelines for the care and use of animals. The experiment was performed in two separate periods. The first group of seven horses was included in the study in November, with three horses given a micro-encapsulated sodium butyrate (Excential Butycoat®, Orffa, Werkendam, the Netherlands) supplemented diet (diet B), and four horses given a placebo (containing only coating material) supplemented diet (diet P), both supplemented at 0.4 g/kg BW. The second experimental period took place in April of the following year, with four horses receiving the butyrate supplemented diet and three horses receiving the placebo supplement. All horses were housed at the same facility and accustomed to the same diet before the start of the study. One week prior to the study all horses were gradually transitioned to the new diet, after which the diets were supplemented with either butyrate or placebo and were fed for 20 consecutive days. The supplement was mixed homogeneously with the concentrate mash, so that concentrate consumption would intentionally lead to supplement intake. A pilot study testing palatability was performed in ponies to ensure voluntary intake of the supplement mixed in a concentrate. Over the course of the present study, feed intake was measured daily along with weekly monitoring of BW, fecal pH and fecal consistency score (1: watery feces, 2: decreased consistency, 3: ideal, 4: hard, 5: constipation). A measuring tape recording girth circumference and body length allowed monitoring of body weight, which was estimated using the equation developed by Carroll and Huntington [23]:

$$ \mathrm{bodyweight}\ \left(\mathrm{kg}\right)=\mathrm{girth}\ {\left(\mathrm{cm}\right)}^2\times \mathrm{length}\ \left(\mathrm{cm}\right)/\mathrm{11,877} $$

After the 20-day period of supplementation, all horses were slaughtered. A penetrating captive bolt gun was used to render the animals unconscious, with exsanguination conducted immediately afterwards to ensure death. Gut content and tissue samples were collected within 30 min post-exsanguination at six different intestinal locations: duodenum (D), jejunum (J), ileum (I), cecum (CAE), left ventral colon (LVC) and right dorsal colon (RDC).

### Assay procedures

**Fig. 1:**
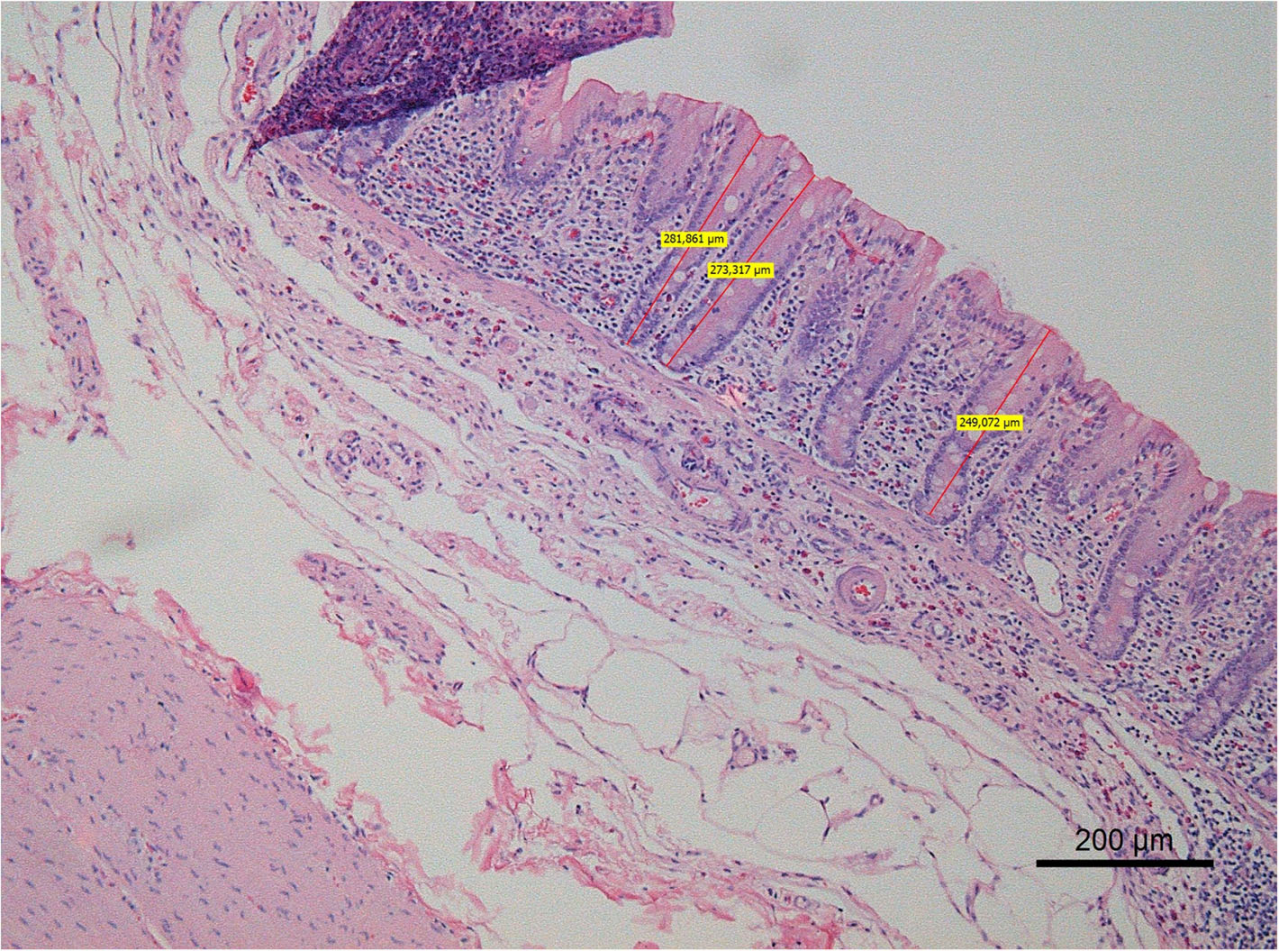
HE staining of the equine cecum, demonstrating crypt depth measurements (μm)

**Fig. 2:**
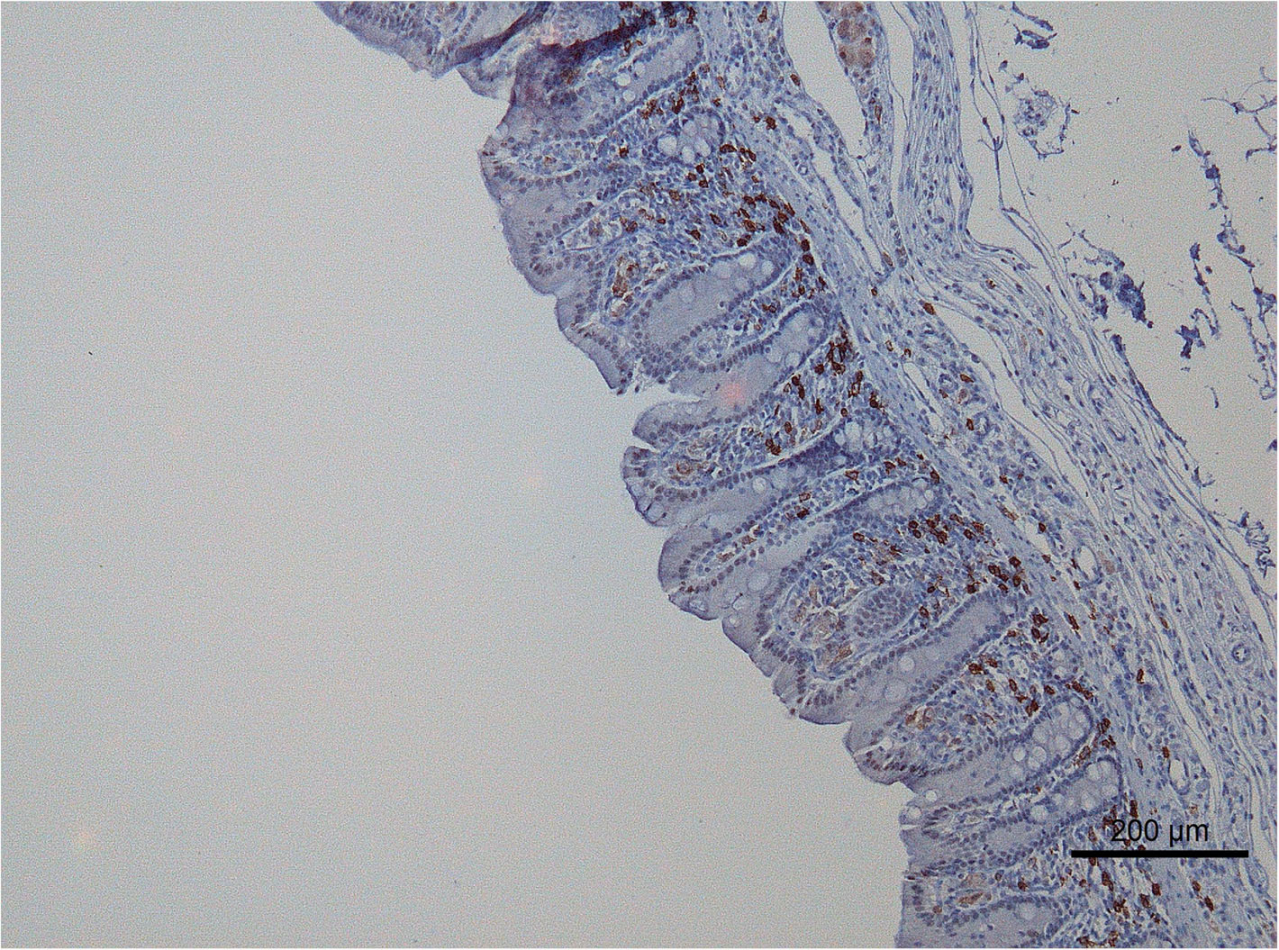
Photomicrograph of CD20 staining of the equine cecum (CD20 positive cells are brown colored)

**Fig. 3:**
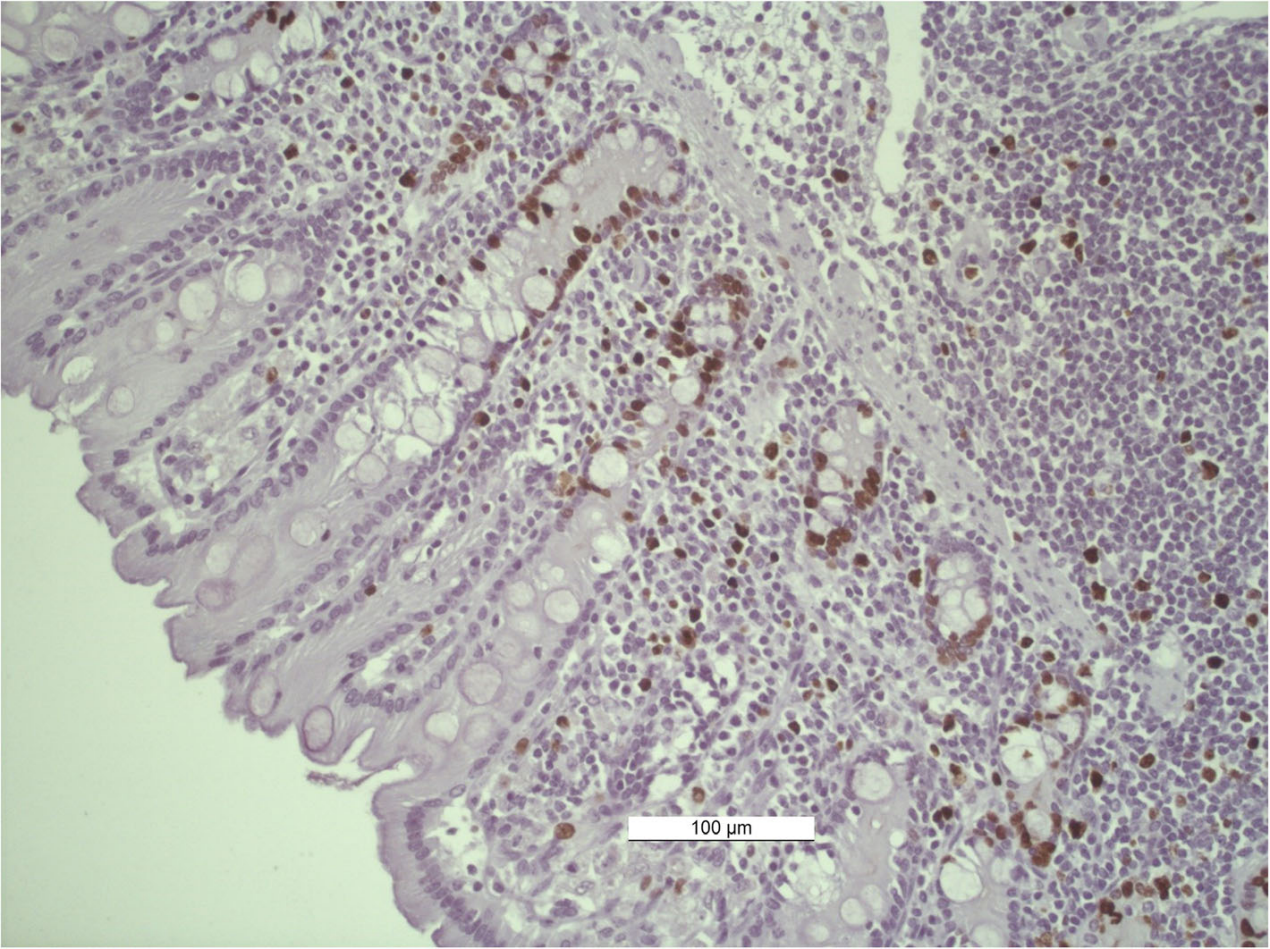
Photomicrograph of KI67 staining of the equine right dorsal colon (KI67+ cells are brown colored)

At each of the intestinal locations, gut content pH was measured in duplicate immediately following collection using a portable pH meter (Hanna Instruments, Temse, Belgium). Gut tissue samples (2x2cm) were initially preserved in formaldehyde and further processed after 24 h. After dehydration of the formalin-fixed samples in xylene, the tissue blocks were trimmed for embedding in paraffin, taking care to orientate the tissue in such a way that the full length of crypts and villi would be visible in the tissue sections. Sections of 4 mm thickness were cut with a Microm microtome (Prosan, Merelbeke, Belgium) and mounted on glass slides. After deparaffination in xylene (2 times, 5 min), the tissue sections were rehydrated in isopropylene (5 min), 95% ethanol (5 min) and 50% ethanol (5 min). Sections were stained with haematoxylin and eosin (H&E) (Fig. 1). Immunohistochemical staining for CD3, CD20 and KI67 positive (for the RDC sections) cells was performed utilising different sections obtained as previously described. CD3 cell staining is characterized as a immunohistochemical marker for T-cells in tissue sections and CD20 staining (Fig. 2) as a marker for B cells [24]. Staining of KI67 positive cells (Fig. 3) allows evaluation of proliferating cells [25]. Sections were examined using a DM2000 microscope and a Leica Camera DFC320 (Leica Microsystems Ltd., Wetzlar, Germany) coupled to a computer-based image analysis system LAS® (v.3.8., Leica Microsystems Ltd). Villus length and crypt depth were measured on the H&E stained sections, measuring three villi and three crypts per sample. CD3 and CD20 cell density in the lamina propria was measured using “area percentage” from three locations per sample. KI67 positive cells were counted individually in nine crypts per RDC sample.

### Statistical methods

Prior to further analysis, normality of distribution was confirmed for all data using the Kolmogorov-Smirnov and Shapiro-Wilk tests and equality of data variability was checked with Levene’s test. For these tests, Superior Performing Software Systems version 23 (SPSS Inc., Chicago, Illinois, USA) was used. To evaluate the effect of butyrate supplementation on feed intake, BW, fecal pH and fecal consistency, a longitudinal analysis was performed using R-studio® (version 3.2.5, 16 April 2016) and the packages ‘gplot’, ‘lattice’, ‘ggplot2’ and ‘nlme’. All other data were analysed in a general additive mixed model with treatment (butyrate versus placebo), period, age, sex and treatment x period as fixed effects and horse as random effect. These analyses were conducted using R-studio® (version 3.2.5, 16 April 2016) and package ‘gamm4’. *P*-values below 0.05 were set as statistically significant and a *P* value between 0.1 and 0.05 was defined as a trend.

## Data Availability

The datasets used and/or analysed during the current study are available from the corresponding author on reasonable request.

## Abbreviations

BCS: Body condition score
BW: Body weight
Diet B: Butyrate supplemented diet
Diet P: Placebo supplemented diet
D: Duodenum
I: Ileum
J: Jejunum
CAE: Caecum
LVC: Left ventral colon
RDC: Right dorsal colon
H&E: Haematoxylin and eosin
DM: Dry matter
